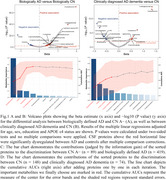# Multiplex cerebrospinal fluid metabolomics identifies biomarkers for diagnosis and prediction of Alzheimer's disease

**DOI:** 10.1002/alz70856_101442

**Published:** 2025-12-25

**Authors:** Jiaming Lu, Qian Chen, Yajing Zhu, Futao Chen, Bing Zhang

**Affiliations:** ^1^ Department of Radiology, Nanjing Drum Tower Hospital, Affiliated Hospital of Medical School, Nanjing University, Nanjing, Jiangsu, China; ^2^ Nanjing Drum Tower Hospital, Affiliated Hospital Clinical College of Nanjing Medical University, Nanjing, Jiangsu, China

## Abstract

**Background:**

Recent expansion of metabolomic coverage opens unparalleled avenues to unveil new biomarkers of Alzheimer's disease (AD).

**Method:**

We included 635 participants from the Alzheimer's Disease Neuroimaging Initiative (ADNI) with baseline clinical diagnoses of CN (23.15%), MCI (56.69%) and AD dementia (20.16%). All included participants not only underwent comprehensive CSF metabolomic assays but also had complete CSF Aβ42. 419 A+ (including 58 preclinical AD, 241 MCI due to AD and 120 dementia due to AD) participants were biologically defined as AD. 172 CN participants from the Parkinson's Progression Markers Initiative (PPMI) cohort were also enrolled.

**Result:**

Among 348 cerebrospinal fluid (CSF) metabolite analysed from the ADNI database, the combination of N‐acetylthreonine and choline performed best in diagnosing both biologically (AUC = 0.961) and clinically (AUC = 0.833) defined AD. Four‐ (*N*‐acetylthreonine, choline, N‐acetylserine and 2‐O‐methylascorbic acid) and four‐ (*N*‐acetylthreonine, choline, O‐sulfo‐L‐tyrosine and 3‐amino‐2‐piperidone) metabolite panels greatly improved the accuracy to 0.987 and 0.871, respectively. Their superior performance was validated in an independent external cohort. Moreover, they effectively predicted the clinical progression to AD dementia and were strongly associated with AD core biomarkers and cognitive decline.

**Conclusion:**

Our findings revealed promising high‐performance biomarkers for AD diagnosis and prediction.